# Effect on work ability and health-related quality of life following an interactive patient education aiming to increase sense of coherence and health literacy – the LEARN-to-COPE cluster randomized trial

**DOI:** 10.1080/02813432.2025.2507859

**Published:** 2025-05-22

**Authors:** Märit Löfgren, Lena Nordeman, Nashmil Ariai, Cecilia Björkelund, Gun Rembeck, Irene Svenningsson, Karin Törnbom, Dominique Hange

**Affiliations:** a Primary Health Care/School of Public Health and Community Medicine, Institute of Medicine, The Sahlgrenska Academy, University of Gothenburg, Gothenburg, Sweden; b Research, Education, Development & Innovation Primary Health Care, Research, Education, Development & Innovation Center Södra Älvsborg, Region Västra Götaland, Sweden; c Sahlgrenska Academy, Institute of Neuroscience and Physiology, Sahlgrenska Academy, University of Gothenburg, Gothenburg, Sweden; d Research, Education, Development & Innovation Primary Health Care, Region Västra Götaland, Sweden; e Research, Education, Development & Innovation Primary Health Care, Research, Education, Development & Innovation Center Fyrbodal, Region Västra Götaland, Sweden; f Department of Social Work, University of Gothenburg, Gothenburg, Sweden; g Research, Education, Development & Innovation Primary Health Care, Research, Education, Development & Innovation Center Skaraborg, Region Västra Götaland, Sweden

**Keywords:** Primary health care, sick leave, sense of coherence, health literacy, quality of life

## Abstract

**Objective:**

To evaluate the effect of the LEARN-to-COPE intervention on sick leave, symptoms, and coping.

**Design and setting:**

Cluster-randomized controlled trial including 40 primary care centers (PCCs) in Region Västra Götaland, Sweden. Randomization at the PCC level. Effect of the intervention was compared to Care-as-Usual (CAU). Follow-up was conducted using registry sick leave data, validated questionnaires, and patient-reported data.

**Subjects:**

Primary healthcare patients with recurrent or long-term sick leave or health-related unemployment from included PCCs (*n* = 243).

**Intervention:**

Patient education was conducted via interactive study groups, which convened for half a day every week over eight consecutive weeks. Implementation was centralized in close collaboration with educational associations. The purpose of the intervention was to strengthen participants’ sense of coherence and health literacy, with the aim of enhancing their work ability and health.

**Main outcome measures:**

The primary outcome measure was change in scheduled activity, derived from data on sick leave (obtained from the Swedish Social Insurance Agency) and participation in work-oriented rehabilitation (self-reported occupational status). Secondary outcomes (symptoms and coping) were measured with validated questionnaires at baseline and follow-ups after 3, 6, and 12 months.

**Results:**

Included participants suffered from anxiety, depression, exhaustion, and pain and had poor health-related quality of life. After 12 months, there was no significant change in scheduled activity, sense of coherence, symptoms, or health-related quality of life, but a statistically significant positive change in health literacy and self-efficacy was found in the intervention group.

**Conclusion:**

Considering participants’ pronounced burden of symptoms, the focus should be on improving the sick leave process as a whole, rather than seeking quick remedies for patients’ complex health issues. Centralized implementation of the intervention was a promising concept that deserves further evaluation.

**Trial registration number:**

Clinicaltrials.gov NCT04254367.

## Introduction

The societal costs related to long-term health-related reduced work ability and sickness absence are extensive, and trends show that these costs are increasing [1]. The largest diagnostic groups among sick-listed patients in Sweden are common mental disorders (including depression, anxiety syndromes, and stress-related disorders such as adjustment disorder/exhaustion disorders), accounting for 51% of patients, and musculoskeletal and connective tissue disorders (including nonspecific musculoskeletal pain), accounting for 16% of patients. Together, these conditions comprise two out of three sick leave cases exceeding 90 days, corresponding to nearly 100,000 concurrent ongoing cases in Sweden [2]. By comparison, significantly fewer individuals, 69,000 persons, were diagnosed with cancer over an entire year [3].

The sick leave and rehabilitation process (SRP) encompasses efforts by healthcare, the Swedish Social Insurance Agency, and other relevant stakeholders to support individuals with reduced work ability in returning to work or other meaningful activities, with or without lasting functional impairments [4].

Patients in the SRP often describe severe impairments in work ability due to their health conditions, making return-to-work attempts seem impossible [5–7]. Their work capacity is further reduced by medication side effects and the challenge of attending health appointments during work hours [5]. Additionally, the patients may suffer setbacks after overexerting themselves on better days [6], leading them to fear emotional outbursts and avoid certain situations [7]. In Sweden, patients in the SRP perceive a gap between their care expectations and the SRP services provided, citing deficiencies in coordination, care transitions, and person-centeredness [8].

A systematic review of previous intervention studies targeting SRP outcomes evaluated the effects of unimodal or multimodal interventions involving different professionals (physiotherapists, psychotherapists, occupational therapists, case workers at the Social Insurance Agency), and different interventions (such as therapeutic interventions, rehabilitation coordination, case management, and work-oriented rehabilitation) [9]. Compiled results showed that interventions involving coordination activities or workplace contact may positively impact short-term return to work (RTW) rates for individuals on sick leave due to mental or musculoskeletal disorders. Still, the evidence regarding this remains uncertain [9].

Notably, most prior SRP intervention studies were either constructed from the perspective of one profession (physiotherapy, cognitive behavioral therapy, nurse care management), or one SRP actor (such as primary healthcare, the Social Insurance Agency, occupational health services, or hospital clinics). Further, most studies were limited to specific health conditions or specified periods of sick leave duration, or they excluded patients on long-term sick leave exceeding 6-12 months or unemployed patients. However, some intervention studies involved integrated care that could include multiple healthcare professions, a combination of medical and vocational rehabilitation, team-based assessments, or collaboration with case workers outside the healthcare system. Overall there are too few SRP intervention studies incorporating multiprofessional and multi-organizational perspectives while addressing patients’ long-term, complex health problems.

Sense of coherence—perceiving life as comprehensible, manageable, and meaningful—is suggested to protect against the adverse effects of external stressors like illness [10]. A reduced sense of coherence in patients with persistent physical symptoms and long-term sick leave is associated with frequent healthcare utilization [11], poor adjustment to illness and a higher likelihood of comorbidity with depression [12], and prolonged sick leave [13].

Health literacy encompasses the skills needed to engage in healthcare decisions and make informed choices. These skills include accessing and understanding health information, critically evaluating it, and possessing the communication and social abilities for person-centered health dialogues and decision-making [14–16]. Enhanced health literacy is associated with better health outcomes, lower healthcare costs, and reduced healthcare utilization [17–19]. Health literacy develops through cognitive and psychosocial growth, formal education, lifelong learning, and personal experiences [15].

Unlike previously studied interventions, the LEARN-to-COPE intervention was neither constructed from the perspective of a single profession nor the standpoint of just one SRP actor. Additionally, the focus of the LEARN-to-COPE was not on treatment, rehabilitation, assessment, or improved coordination of care. Instead, the purpose of the intervention was to improve participants’ health literacy and sense of coherence with the aim of improving their ability to influence their health and work ability [20].

Prior research findings highlighted that improved health is not necessarily associated with work participation [21]. The underlying causes are insufficiently studied, but non-medical factors may impact the outcome of the sick leave and rehabilitation process (SRP) [22,23].

Patients with very low ability to engage in activities often start with work-oriented rehabilitation during sick leave before returning to work, which is a step closer to the labor market. Additionally, work placements provide information about individual work-related abilities and difficulties and can serve as a decision basis for either continued focus on rehabilitation efforts or early retirement [23]. There is a lack of studies examining increased participation in work-oriented rehabilitation and intervention effects in patients with combined mental and musculoskeletal disorders. Similarly, few studies have examined long-term impaired work ability in a primary healthcare context.

The purpose of this study was to evaluate the effects of the LEARN-to-COPE intervention on changes in scheduled activity (work or work-oriented rehabilitation), symptoms, and coping. The hypothesis was that the intervention would strengthen sense of coherence and health literacy, entailing a positive change in scheduled activity and symptoms compared to Care-as-Usual (CAU). A previous pilot study of LEARN-to-COPE showed promising results [20].

## Materials and methods

### Trial design

This study employed a quantitative, pragmatic, cluster-randomized controlled trial design with two arms (intervention arm and control) to evaluate the LEARN-to-COPE intervention. Each included primary care center (PCC) was treated as a cluster. The cluster design was used to allow for the pragmatic implementation of the study by clustering intervention participants in time and location. Study objectives and outcome measures pertained to the individual participant level.

The inclusion of PCCs and patients was made according to the Intention to Treat. The effect of the LEARN-to-COPE intervention in addition to CAU was compared to CAU. The primary outcome measure was change in scheduled activity, derived from data on sick leave (obtained from the Swedish Social Insurance Agency) and participation in work-oriented rehabilitation (self-reported occupational status). Secondary outcomes included health literacy, sense of coherence, health-related quality of life, symptoms of mental illness and pain, lifestyle habits, and self-efficacy. Additionally, contextual data such as quality of care, patient-reported experiences with the intervention, and LEARN-to-COPE-specific outcomes were monitored. Follow-up was performed using registry sick leave data, validated questionnaires, and patient-reported contextual data at baseline and after 3, 6, and 12 months. The H0 hypothesis was that there was no difference in outcomes between the intervention and control arms. The study was preregistered in ClinicalTrials.gov, Study ID NCT04254367.

### Setting and cluster eligibility

This study was conducted between 2019 and 2022 in the primary healthcare context of Region Västra Götaland, Sweden, with 201 PCCs, half of which were publicly operated and half privately owned. To avoid interference from other ongoing major studies on mental health and sick leave, we selected PCCs not participating in those studies. Additionally, only PCCs with rehabilitation coordinators were included, as these coordinators served as local contacts responsible for coordinating participant selection.

### Study population

The study targeted adult primary healthcare patients of working age (18–64 years) with long-term or recurrent health-related sick leave or unemployment, independently of diagnosis. Inclusion and exclusion criteria were as follows:

**Table:** 

Inclusion criteria	Exclusion criteria
Adult patients of working age (18–4 years) Health-related sick leave or unemployment >60 net days during the last six months	Patient is expected to return to work or job-seeking following an existing healthcare and rehabilitation plan Serious mental disorder needing psychiatric specialist care or acute crisis response Somatic disease a definite barrier to rehabilitation for a foreseeable future Cognitive impairment or not speaking/understanding Swedish

Local rehabilitation coordinators and GPs, in dialogue with each individual patient, assessed if patients were expected to return to work or job-seeking. Their assessments were guided by whether a consensus could be reached on a realistic, individualized plan to address both medical and non-medical barriers to return to work (RTW). Patients who were already successfully adhering to an existing RTW plan were excluded from the study.

### Intervention

The LEARN-to-COPE intervention, the rationale of the intervention, and related procedures have been described in detail in a previous pilot study [20]. In short, the intervention consisted of interactive patient education within supervised study groups alongside CAU.

The sessions, designed to accommodate 6–12 participants, convened for half a day every week over eight consecutive weeks, following a structured study plan. The sessions addressed physiological reactions to stress and chronic pain, the impact of lifestyle habits on well-being, sources of evidence-based health information, SRP regulations and available support, and tools and communication strategies for practical problem solving to enhance participants’ health literacy. Additionally, participants discussed and reflected on how external factors, their own communication methods, and personal motivations influenced their sense of coherence in a personal context.

The intervention comprised various elements that could potentially improve patient well-being and workability: (i) the educational content, (ii) the pedagogic method with interactive learning and peer support, (iii) enhancement of health literacy and/or a stronger sense of coherence, (iv) the support network provided by primary healthcare, case managers, and employers to facilitate RTW from a person-centered perspective.

The intervention targeted individuals and was delivered through study groups held in facilities located near each PCC. The research team recruited seven study group supervisors to facilitate discussions using a manual developed within primary healthcare by a GP, a psychologist, and an occupational therapist, with input from one of the supervisors and a patient representative. Although they did not have medical training, the supervisors had prior experience in group coaching, teaching communication skills within business settings, and leading community initiatives geared toward the target population. When necessary, the supervisors could consult the first author, a GP with no prior relationship to the participants, for guidance. The first author was occasionally consulted to provide a medical perspective on questions raised within the study groups. However, if participants had personal medical concerns, the supervisors directed them to their PCC instead.

### Comparison

Patients in the control group received CAU according to local procedures, which included all relevant medical, psychological, and psychoeducational treatments.

### Demographic data

Collected demographic data included age, gender, marital status, educational level, current occupation, country of birth, registered sick leave, occupation, and employment status.

Age and gender were retrieved from participants’ personal identity numbers. Sick leave data was collected from the MicroData for Analysis of Social insurance (MiDAS) database. Marital status, educational level, current occupation, country of birth, and duration of health-related impaired workability for unemployed participants were patient reported.

Employment status was determined according to the Statistics Sweden socio-economic classification system, known as ‘Socioeconomic indexation’ (SEI) [24]. Self-reported job titles from questionnaire data were categorized into three groups: (1) High white collar, (2) Middle/low white collar, and (3) Blue collar/students [25].

### Outcomes

Primary Outcome Measure was *change in scheduled activity (work or work-oriented rehabilitation)*. Scheduled working hours were assessed based on registry sick leave data when available (gross and net sick leave days and start/end dates of sick spell/early retirement), combined with self-reported data about work status/working hours, sick leave, and participation in scheduled rehabilitation. Registry data and self-reported data were juxtaposed for each participant manually to analyze changes in scheduled activities: returned to work full-time/part-time/with reduced working hours without sickness benefit, became unemployed/an early retiree/a student, participated in medical or work-oriented rehabilitation, received a protected employment, or no change/less active. Individual change in activity level (scheduled hours per week) was dichotomized as increased activity level Yes/No. This procedure differed from the original plan to compare hours of activity, as the exact hours of work-oriented rehabilitation were often unclear in the data.

Secondary outcomes were perceived work ability, sense of coherence, health literacy, health-related quality of life, symptoms of mental illness and pain, self-assessed change in activity level, lifestyle habits, self-efficacy, quality of care, patient-reported experiences, and LEARN-to-COPE-specific outcomes.

*Perceived work ability* was measured with the Work Ability Score (WAS) VAS scale [26,27], *sense of coherence* was measured with the Sense of Coherence Scale-13 (SOC-13) [10], and *health literacy* was measured with the European Health Literacy Survey Questionnaire, 16-item version, Swedish edition (HLS-EU-Q16-SE) [28]. *Health-related quality of life* was measured with the EuroQol 5-Dimension (EQ-5D) [29], which measures five dimensions of health-related quality of life independently of health condition: mobility, self-care, usual activities, pain/discomfort, and anxiety/depression. Symptoms of mental illness, including *depression, exhaustion,* and *anxiety,* were measured with the Montgomery-Åsberg Depression Rating Scale - Self-rated version (MADRS-S) [30], the Karolinska Exhaustion Disorder Scale (KEDS) [31], and the Generalized Anxiety Disorder 7-item Scale (GAD-7) [32]. *The risk of future disability and long-term sick leave because of pain* was assessed with the Örebro Musculoskeletal Pain Screening Questionnaire (ÖMPSQ) [33], prevalence of *widespread pain,* defined as pain registered in both sides of the body, above and below the waist, and in the axial skeleton [34], was assessed using predefined body regions [35], and *pain catastrophizing*, a contributing factor to long-term debilitating pain, was measured with the Pain Catastrophizing Scale (PCS) [36]. Ranges, subscales, and guides to interpretation for these scales are presented in Table 1.

**Table 1.** Ranges, subscales, and guides to interpretation for validated scales used in the LEARN-to-COPE cluster randomized controlled trial.

Outcome variable	Validated scale	Range	Subscales (range)	Guide to interpretation
Perceived work ability	Work Ability Score (WAS) VAS scale	0–10 Points		A higher value indicates better work ability
Sense of coherence	Sense of Coherence Scale-13 (SOC-13)	13–91 Points	Comprehensibility (5–35 points) Manageability (4–28 points) Meaningfulness (4–28 points)	A higher value indicates higher sense of coherence
Health literacy	European Health Literacy Survey Questionnaire, 16-item version, Swedish edition (HLS-EU-Q16-SE)	0–16 Points		0–8 Points indicate inadequate, 9–12 points problematic, and 13–16 sufficient health literacy
Health-related quality of life	EuroQol 5-Dimension (EQ-5D)	−0.594 − 1		Index score of +1 indicates complete health, an index score of 0 equals death, and values less than 0 indicate health states worse than death
Symptoms of depression	Montgomery-Åsberg Depression Rating Scale - Self-rated version (MADRS-S)	0–54 Points		0–12 Points indicate no depression, 13–19 points mild depression, 20–34 points moderate depression, and scores above 34 points indicate severe depression
Symptoms of exhaustion	Karolinska Exhaustion Disorder Scale (KEDS)	0–54 Points		Values above 18 points indicate stress-related exhaustion syndrome
Symptoms of anxiety	Generalized Anxiety Disorder 7-item Scale (GAD-7)	0–21 Points		0–4 Points indicate no or minimal anxiety, 5–9 points mild Generalized Anxiety Disorder (GAD), 10–14 points moderate GAD, and ≥15: severe GAD
The risk of future disability and long-term sick leave because of pain	Örebro Musculoskeletal Pain Screening Questionnaire (ÖMPSQ)	0–100 Points		Values above 50 indicate an increased risk of future disability and long-term sick leave because of pain
Widespread pain	Predefined body regions			Widespread pain was defined as pain registered in both sides of the body, above and below the waist, and in the axial skeleton
Pain catastrophizing	Pain Catastrophizing Scale (PCS)	0–52 Points	Rumination (0–16 points) Magnification (0–12 points) Helplessness (0–24 points)	A higher value indicates more catastrophizing thought content and numbers above 30 indicate clinical relevance
Sedentary lifestyle	Leisure Time Physical Activity Instrument (LTPAI)			A sedentary lifestyle included extended sitting and minimal exercise (not significantly elevating the breathing rate) less than 4 h per week

*Self-assessed change in activity level* was assessed with the question ‘Have you increased your activity level during current sick spell? Yes/No’.

*Lifestyle habits* were assessed for sedentary leisure-time physical activity with the Leisure Time Physical Activity Instrument (LTPAI) [37]. A sedentary lifestyle was defined as extended sitting or minimal exercise (not significantly elevating the breathing rate) for less than 4 h per week. The difference in Body Mass Index (BMI) change between the intervention and control groups served as a group-level proxy for shifts in eating habits. Response options to the question on smoking included both yes and sometimes. Hazardous alcohol consumption, defined as 3–4 standard drinks (12 grams of pure alcohol) 2–3 times per week or more, was assessed with questions about frequency and volumes.

*Self-efficacy/confidence in own abilities* was assessed with a non-validated 17-item questionnaire inspired by a previous study [38], see Supplementary Information 1. Questions regarded confidence in own knowledge and ability to maintain activities, solve problems, and alleviate symptoms of ill health. All responses were provided on a 5-level Likert scale that ranged from not at all true to completely true, with 1–5 points per item and a total sum of 85 points.

*Quality of care from a patient perspective* was assessed with a non-validated study-specific 20-item questionnaire inspired by values intrinsic to person-centered care, previous research [38], and interviews with SRP stakeholders, see Supplementary information 2. Responses were provided on a 5-level Likert scale that ranged from not at all true to completely true. Topics included healthcare personnel and case managers being respectful and committed (eight items, range 8–40 points), receiving person-centered information (seven items, range 7–35 points), involvement in healthcare (three items, range 3–15 points), and relationship continuity (two items which were dichotomized and analyzed separately). For the purpose of analysis, the items were also dichotomized: very true/completely true = very true, and not at all true/only partly true/moderately true = not very true.

### Enrollment of PCCs

PCCs were contacted by phone and e-mail. After receiving information about the study, but before randomization, the head of the PCC signed a written consent to participate. After that, enrolled PCCs each received a study start-up visit from the research group (first author), aiming at informing employees involved in the local sick leave and rehabilitation process (GPs, the rehabilitation coordinator, and any psychosocial team members) about the research study, the intervention, and inclusion/exclusion criteria. The researcher was blinded to the PCC allocation at these meetings.

The PCCs were enrolled in four batches of 2–16 PCCs from autumn 2019 to autumn 2021. Randomization was performed at the PCC level within each batch. Enrolled PCCs were stratified into two groups, rural and urban, before randomization. An independent statistician used computer-generated tables to allocate study arms in a 1:1 ratio within each stratum.

### Enrollment of patients

Patients from each PCC (both arms in each batch) were enrolled a few weeks before the planned start date of the intervention groups for each batch. Included PCCs remained blinded to allocation during the selection process.

Local primary healthcare professionals at the cluster level, such as rehabilitation coordinators and GPs, selected patients for inclusion based on the inclusion and exclusion criteria. Professionals involved in the patients’ SRP, who were informed about the intervention, study, and criteria, were encouraged to engage in dialogue to identify those who were lacking consensus concerning a realistic, individualized plan for sick leave and rehabilitation that would support their return to work.

The healthcare personnel asked potential participants if they consented to receive more information about the research study. A research assistant then contacted them to provide written and oral information about the study and answer their questions. The patients knew if their PCC was randomized to intervention or control when providing their written consent to participate.

### Data collection

Data was collected between 2019 and 2022. Data on sickness benefit days was retrieved from the MicroData for Analysis of Social insurance (MiDAS) database, covering the period from one year before baseline to one year after baseline. Questionnaires were distributed *via* secured web links and stored in esMaker (Entergate AB), linked to the researcher’s account on the Region Västra Götaland’s protected network. The questionnaires were administered at baseline and at 3, 6, and 12 months follow-up. A research assistant managed distribution and reminders.

### Statistical analysis

Research subject identities were replaced with individual codes before the analysis. Survey responses were compiled and interpreted according to the instructions provided in each survey’s manual.

Statistical methods were employed for descriptive analysis depending on data level. Continuous variables were analyzed using t-tests for two independent groups or the Mann-Whitney U test. Categorical variables were analyzed using Pearson’s Chi-Square Test. Linear mixed model analysis was performed to control for correlation and changes in repeated measures for each ‘person’ and ‘center’ and cluster randomization of PCC. These analyses were used to compare means of individual changes between the intervention group and the control group in all continuous(scale) variables and were adjusted for cluster randomization at the PCC level and for repeated measures within individuals. The models included a repeated effect for ‘time’ for each ‘person’ and ‘center’ and a random intercept for each ‘center’. Linear mixed model analysis was also used for comparison of total number of 12 months net sick-leave days between intervention and control group and were adjusted for cluster randomization of PCC by including a random intercept for each ‘center’ in the model. EMMEANS subcommand from Mixed model in SPSS was used to compare estimated marginal means between the intervention group and the control group for every time in the study. Logistic regression analysis was done to compare ‘increased activity from baseline’ between the intervention and control group. All analyses were adjusted for the pre-specified variables age, sex, antidepressants, and addictive drugs on prescription at inclusion. Logistic regression analysis was also controlled for the net number of sick leave days last year before inclusion.

**Table 5.** Outcome variables at 12 months follow-up for participants in the LEARN-to-COPE cluster randomized controlled trial.

Outcome variable	Total *n* = 243	Control *n* = 142	Intervention *n* = 101	*p*-Value
Net sick leave days from baseline to 12 months follow-up (registry data), mean* (95% CI)		255 (225–286)	218 (181–254)	0.12
Change in work status**, *n* (%)				
RTW (full-time or part-time)	54 (25.0)	27 (20.5)	27 (32.1)	0.15
Begun work-oriented rehabilitation or studying	19 (8.8)	10 (7.6)	9 (10.7)	
Begun medical rehabilitation	5 (2.3)	4 (3.0)	1 (1.2)	
Waiting for rehabilitation to start	52 (24.1)	32 (24.2)	20 (23.8)	
No change or less scheduled activity	40 (18.5)	24 (18.2)	16 (19.0)	
Early retirement	4 (1.9)	4 (3.0)	0 (0.0)	
Lost sickness benefit or became unemployed full-time or part-time	42 (19.4)	31 (23.5)	11 (13.1)	
Increased their scheduled (work or work-related) weekly hours**, *n* (%)	75 (36.6)	39 (30.7)	36 (46.2)	0.40
Participation in any rehabilitation (self-reported data), *n* (%)	36 (38.3)	21 (35.6)	15 (42.9)	0.48
Perceived work ability, mean* (95% CI)		3.7 (2.9–4.5)	4.4 (3.5–5.4)	0.21
Sense of coherence, mean* (95% CI)		52.0 (48.2–55.9)	55.3 (50.6–59.9)	0.27
Health literacy, mean* (95% CI)		12.9 (12.1–13.8)	14.2 (13.2–15.2)	**0.049**
Self-efficacy/confidence in own abilities, mean* (95% CI)		48.9 (45.0–52.8)	55.2 (50.4–60.0)	**0.039**
Health-related quality of life, mean* (95% CI)		0.39 (0.32–0.47)	0.39 (0.29–0.48)	0.91
Symptoms of mental illness				
Depression, mean* (95% CI)		20.8 (18.1–23.5)	17.5 (14.2–20.8)	0.11
Exhaustion, mean* (95% CI)		35.2 (32.6–37.9)	32.3 (29.0–35.5)	0.14
Anxiety, mean* (95% CI)		15.6 (14.1–17.1)	14.0 (12.2–15.9)	0.17
Pain				
Risk of long-term sick leave due to pain, *n* (%)	72 (52.2)	46 (52.9)	26 (51.0)	0.83
Widespread pain, *n* (%)	62 (36.7)	38 (36.2)	24 (37.5)	0.86
Pain catastrophizing >30, *n* (%)	37 (21.9)	24 (22.9)	13 (20.3)	0.70
Pain catastrophizing, mean* (95% CI)		20.5 (17.2–23.9)	21.1 (17.1–25.2)	0.81
Lifestyle habits				
Sedentary lifestyle, *n* (%)	39 (23.1)	25 (23.8)	14 (21.9)	0.80
Alcohol high, *n* (%)	4 (2.4)	4 (3.8)	0 (0.0)	0.11
Smoking (yes + sometimes), *n* (%)	28 (16.6)	18 (17.1)	10 (15.6)	0.78
Obesity, BMI ≥30, *n* (%)	61 (36.7)	45 (44.1)	16 (25.0)	0.13
BMI, mean* (95% CI)		30.0 (28.5–31.6)	28.0 (26.2–29.8)	0.083
Quality of care				
Healthcare personnel and case managers being respectful and committed, mean*** (SD) Person-centered information, mean*** (SD) Involvement in healthcare, mean*** (SD) GP continuity good/very good, *n* (%) Rehabilitation coordinator continuity good/very good, *n* (%)	27.8 (7.9) 21.8 (7.0) 2.1 (0.8) 90 (53.3) 75 (44.4)	28.2 (8.0) 21.6 (7.4) 2.2 (0.8) 63 (60.0) 53 (50.5)	27.1 (7.7) 22.1 (6.5) 2.1 (0.9) 27 (42.2) 22 (34.4)	0.37 0.63 0.74 **0.024** **0.041**

Missing values not included. Statistically significant changes in health literacy and self-efficacy favored the intervention arm. Participants in the intervention group reported lower quality of care, which may have compromised the intervention′s effectiveness.

Bold text: statistically significant difference between intervention and control group (*p* < 0.05); Analyses were conducted using Pearson’s Chi-Square Test unless otherwise specified; *indicates adjusted means from linear mixed model analysis; **combined registry and self-reported data were used to determine participants’ weekly hours of scheduled work or work-related rehabilitation, including both employed and unemployed individuals. The difference in weekly hours between the 12-month follow-up and the baseline was calculated, and the resulting variable was dichotomized based on whether there was an increase in scheduled hours.; ***analysis performed using *t*-tests for two independent groups; Registry data was retrieved from the MicroData for Analysis of Social insurance (MiDAS) database; Perceived work ability: Work Ability Score (WAS) VAS scale; Sense of coherence: Sense of Coherence Scale-13 (SOC-13); Health Literacy: European Health Literacy Survey Questionnaire, 16-item version, Swedish edition (HLS-EU-Q16-SE); Self-efficacy/confidence in own abilities: study specific questionnaire range 17–85 points, a higher value indicates better self-efficacy; Health-related quality of life: EuroQol 5-Dimension (EQ-5D); symptoms of depression: Montgomery-Åsberg Depression Rating Scale – Self-rated version (MADRS-S); symptoms of exhaustion: Karolinska Exhaustion Disorder Scale (KEDS); symptoms of anxiety: Generalized Anxiety Disorder 7-item Scale (GAD-7); The risk of future disability and long-term sick leave because of pain: Örebro Musculoskeletal Pain Screening Questionnaire (ÖMPSQ); Widespread pain: pain registered in both sides of the body, above and below the waist, and in the axial skeleton; Pain catastrophizing: Pain Catastrophizing Scale (PCS); A sedentary lifestyle: extended sitting and minimal exercise (not significantly elevating the breathing rate) less than 4 h per week: 3-4 standard drinks (12 grams of pure alcohol) 2-3 times per week or more; Smoking: both yes and sometimes; BMI: body mass index.

The significance level was set at *p* < 0.05. Data analysis was performed using SPSS version 29 and reported following the CONSORT checklist for cluster randomized trials [39]. All researchers were blinded to allocation until the analyses were complete.

### Non-participants

Despite multiple attempts to reach participants by mail and telephone, a total of 19 patients were lost to follow-up at baseline and all subsequent measurement points, despite not formally withdrawing from the study. No statistically significant differences were found between participants and non-participants in terms of sex or age (the only available data). Available survey data and registry data were analyzed for participants who did not actively withdraw their consent to participate.

### Power calculation

A power calculation was performed, assuming that 16 PCCs per treatment group, with 7 patients per PCC (thus 112 subjects per treatment group), will have a power of almost 80% and a significance of 5%, using a two-sided test to detect a difference of 20 days of sick leave, with a variance of 32 days and an intercluster correlation coefficient of 0.3. We did not adjust for the attrition rate.

## Results

### The selection process, included PCCs, and data availability

**Figure 1.**
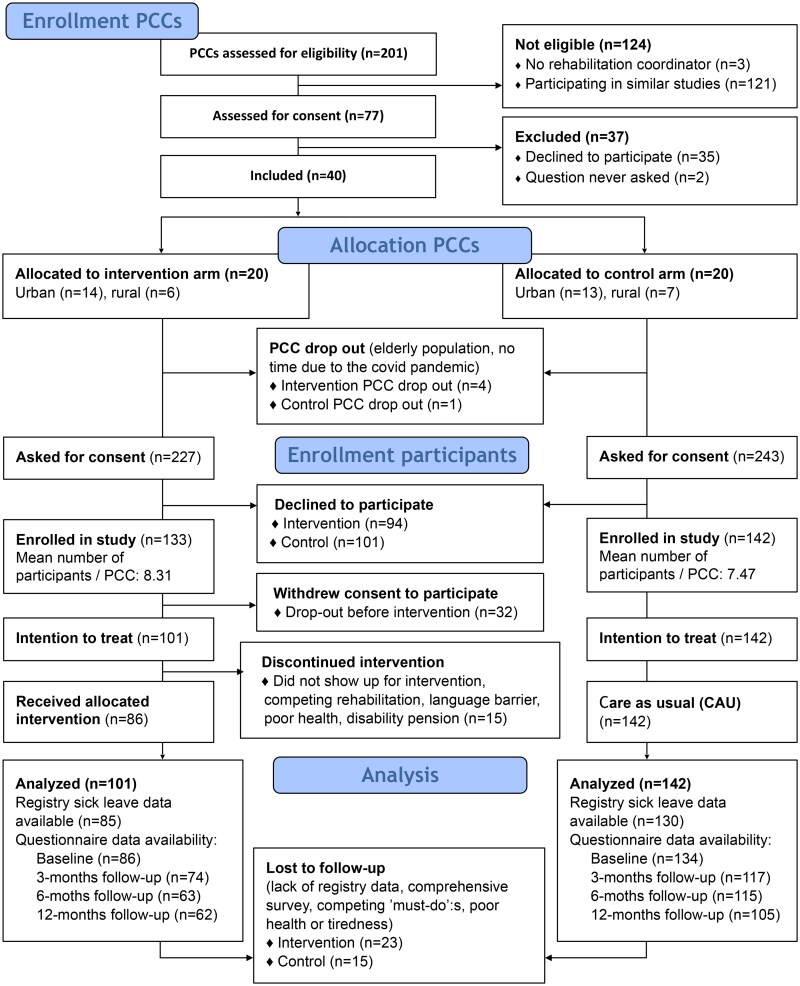
Consort flow chart of the LEARN-to-COPE cluster randomized trial. Each included primary care center (PCC) was treated as a cluster.

The selection process and data availability are illustrated in the study’s CONSORT Flow Diagram [40], see Figure 1.

Seventy-seven PCCs were assessed for consent, and about half of them (*n* = 40) were included. Most PCCs (*n* = 32) were enrolled in October 2019 – February 2020 for planned study start in February 2020 to April 2020 (the first two batches). Enrolling PCCs was unproblematic before March 2020 (outbreak of the COVID-19 pandemic) and slow thereafter. The last PCCs were enrolled in spring 2021.

Characteristics of included PCCs are detailed in Table 2. Stratification of PCCs before randomization secured equal distribution of urban/rural PCCs in each study arm. Distribution of sizes and the Care Need Index were similar in the two arms, but there were more publicly governed PCCs in the control group. Five PCCs dropped out after randomization, four intervention PCCs and one control PCC. Reasons for drop-out were few eligible patients (elderly population, >64 years), heavy workload within primary healthcare in general, and heavy workload related to the COVID-19 pandemic specifically.

**Table 2.** Characteristics of included primary care centers (PCCs) in the LEARN-to-COPE cluster randomized controlled trial.

Allocation & batch #	Number of PCCs	Urban/rural	Private/public	Size (min–max)	Care Need Index	Attrition
Intervention total	20	14/6	11/9	2300–18,500	1.7 − 5.2	4
Batch 1	*8*	*5/3*	*5/3*	*3300 − 10,500*	*1.7 − 5.2*	*–*
Batch 2	*6*	*4/2*	*4/2*	*2300–18,500*	*2.0–5.3*	*1*
Batch 3	*1*	*1/0*	*0/1*	*7500*	*2.3*	*–*
Batch 4	*5*	*4/1*	*2/3*	*3300 − 14,800*	*2.4 − 3.3*	*3*
Control total	20	13/7	7/13	3300 − 17,800	1.5 − 4.9	1
Batch 1	*8*	*5/3*	*4/4*	*3300 − 17,800*	*1.5 − 2.6*	*–*
Batch 2	*6*	*4/2*	*2/4*	*6000–14,000*	*2.0–4.8*	*–*
Batch 3	*1*	*1/0*	*0/1*	*6400*	*1.9*	*–*
Batch 4	*5*	*3/2*	*1/4*	*5600 − 8,700*	*1.9 − 4.9*	*1*
Intervention & control	40	18/22	18/22	2300 − 18,500	1.5–5.2	5

There were more public PCCs in the control group than in the intervention group. Otherwise, no significant differences were observed between the two groups.

The inclusion of participants took place in early January 2020 to September 2021. More than half of patients asked for consent were enrolled in the study in both the intervention (59%) and control (58%) arms. The mean numbers of participants from each PCC were similar in the two arms (intervention 8.31 participants/PCC, control 7.47 participants/PCC). With more intervention PCC dropouts, the control group ended up with more participants.

The baseline was predefined on batch level for both intervention and control arms, except for batch 2. Due to the community spread of COVID-19, it was inappropriate to gather participants in study groups in April 2020, which affected the timeline for batch 2. Enrolled controls followed the original timetable with a baseline in April 2020, while participants in the intervention arm started in September 2020.

In the intervention arm, there was a discontinuation of allocated intervention (*n* = 47) not seen in the control arm. Reasons for discontinuation included parallel rehabilitation interventions, insufficient skills in Swedish, poor health or tiredness, no-show, or ongoing investigation for a disability pension.

Registry sick leave data from the Social Insurance Agency was available for the majority (84% intervention, 92% control). Demographic data was collected only after obtaining written consent, resulting in missing information for participants who consented but did not complete the baseline questionnaire.

Questionnaire response rates declined from 85% in the intervention arm and 94% in the control arm at baseline to 61 and 74%, respectively, after 12 months. Participants’ reasons for not answering were perceptions that the questionnaires were too comprehensive, competing ‘must-do’s, and poor health or tiredness. Notably, some participants who had not answered previous calls returned to answering the subsequent questionnaires. Moreover, registry sick leave data was available for some participants who did not return any questionnaires. Following the intention-to-treat principle, all available data from participants who did not actively withdraw consent was analyzed.

### Participants at baseline

Participant demographic characteristics at baseline (valid percentages) are shown in Table 3. Participants were aged 24–64 years (mean age 47) and predominantly female, with most born in Nordic countries. Educationally, over half had secondary education, one-third held university or college degrees, and a few had only primary education. Socioeconomically, the majority were employed in either high white-collar or low socioeconomic status jobs (i.e. not middle-tier occupations), with approximately two-thirds employed, one-third job-seeking, and less than 1% studying. Nearly 10% were self- or hourly employed. There were no statistically significant differences between the study arms at baseline concerning age, gender, origin, education, or occupation.

**Table 3.** Demographics at baseline for participants in the LEARN-to-COPE cluster randomized controlled study.

Characteristic	Total *n = 243*	Control *n = 142*	Intervention *n = 101*	*p*-Value
Age years mean (SD)	47.4 (9.7)	47.4 (9.7)	46.8 (10.9)	0.50
Gender, *n* (%)				
Women	175 (72.0)	94 (70.7)	65 (75.6)	0.43
Men	68 (28.0)	39 (29.3)	21 (24.4)	
Marital status, *n* (%)				
Married/cohabiting/partnership	149 (68.0)	89 (66.9)	60 (69.8)	0.66
Single/divorced/widow/widower	70 (32.0)	44 (33.1)	26 (30.2)	
Born outside a Nordic country, *n* (%)	19 (8.8)	9 (6.8)	10 (11.8)	0.21
Educational level, *n* (%)				
Up to primary education (nine years of compulsory schooling)	15 (6.8)	8 (6.0)	7 (8.1)	0.70
Secondary education	129 (58.9)	81 (60.9)	48 (55.8)	
University or college	75 (34.2)	44 (33.1)	31 (36.0)	
Working history, *n* (%)				
High white collar	55 (42.3)	28 (37.8)	27 (48.2)	0.37
Middle/low white collar	22 (16.9)	12 (16.2)	10 (17.9)	
Blue collar/students	53 (40.8)	34 (45.9)	19 (33.9)	
Occupation, *n* (%)				
Unemployed	62 (29.5)	36 (29.3)	26 (29.9)	0.49
Studying	1 (0.5)	0 (0.0)	1 (1.1)	
Gainful employed	147 (69.7)	87 (70.7)	60 (69.0)	
Self-employed	6 (2.5)	4 (2.8)	2 (2.0)	0.68
Hourly employed	16 (6.6)	11 (7.7)	5 (5.0)	0.39
Early retirees, *n* (%)	13 (5.3)	10 (7.0)	3 (3.0)	0.16
Net days of early retirement last year, mean (SD)	155.9 (80.5)	140.1 (54.9)	208.8 (140.9)	0.49
Registered sick leave the year before baseline				
Net sick leave days (registry data), mean (SD)	244.1 (110.8)	262.1 (110.4)	214.4 (105.6)	**0.003**
Psychiatric disorders/syndromes and behavioral disorders, *n* (%)	122 (62.2)	71 (58.2)	51 (68.9)	0.22
Musculoskeletal and connective tissue diseases, *n* (%)	36 (18.4)	23 (18.9)	13 (17.6)	
Other diagnoses, *n* (%)	38 (19.4)	28 (23.0)	10 (13.5)	
Duration of impaired workability				
Net sick leave days (registry data), mean (SD)	1214.5 (1010.1)	1348.5 (1061.9)	1009.6 (893.0)	**0.012**
Sick leave >365 days, registry data, *n* (%)	135 (81.8)	86 (87.8)	49 (73.1)	**0.017**
Sick leave >1000 days, registry data, *n* (%)	113 (51.1)	76 (58.0)	37 (41.1)	**0.014**
Health-related unemployment >365 days, *n* (%)	49 (81.7)	30 (83.3)	19 (79.2)	0.68
Medication, *n* (%)				
Antidepressant medication	109 (44.9)	55 (41.4)	54 (62.8)	**0.002**
Recommended drugs long-term pain	56 (23.0)	33 (24.8)	23 (26.7)	0.75
Addictive drugs on prescription	36 (16.4)	28 (21.1)	8 (9.3)	**0.02**

Missing values not included. *p*-Values indicate tests of significant differences between the intervention and control groups. Significant differences were found in sick leave before baseline, use of antidepressant medication, and prescription of addictive drugs.

Bold text: statistically significant difference between intervention and control group (*p* < 0.05); Registry data was retrieved from the MicroData for Analysis of Social insurance (MiDAS) database; Antidepressant medication: non-addictive antidepressive drugs commonly prescribed in primary care (SSRI, SNRI, mirtazapin, mianserin, TCA, and bupropion); recommended drugs long-term pain: pain medications and muscle relaxants prescribed in primary care: paracetamol, NSAID, and chlorzoxazone, anti-depressive or epilepsy drugs prescribed on pain indication: amitriptyline/nortriptyline, duloxetine, and gabapentin; addictive drugs on prescription: opioids, benzodiazepines or benzodiazepine analogues, codeine, or pregabalin. Gabapentin not included.

Most participants experienced long-term impaired workability for over a year, averaging 244 net sick leave days in the year before baseline and a total of 1,215 net sick leave days. Mental disorders were the leading cause of sick leave (62%), followed by musculoskeletal disorders (18%) and other diagnoses (19%). The intervention group had a lower sick leave burden and more participants on antidepressants, whereas the control group had more participants using prescription addictive drugs.

Baseline measurements (valid percentages) are presented in Table 4. Participants exhibited very low perceived work ability. Over half reported no change in their activity levels since their sick leave began, and approximately half engaged in rehabilitation activities, with medical rehabilitation (physiotherapy or psychotherapy) being twice as common as work-oriented rehabilitation.

There were no significant differences between the groups regarding self-reported sick leave, perceived work ability, change in activity level since the beginning of the sick leave, or participation in rehabilitation.

Sense of coherence was low among participants, and half demonstrated inadequate or problematic health literacy. Additionally, participants rated their health-related quality of life as extremely low. On average, participants reported moderate symptoms of depression, pronounced exhaustion, and severe anxiety. Over half were at risk of long-term sick leave due to pain, a significant portion met the criteria for widespread pain, and pain catastrophizing was common. Baseline values for sense of coherence, health literacy, health-related quality of life, symptom scores, and self-efficacy/confidence in one’s own abilities were similar in the two groups.

Smoking and obesity were common, while excessive alcohol consumption was rare. A sedentary lifestyle was more prevalent in the intervention group, with the difference approaching statistical significance. Other lifestyle habits were similar between the two arms.

### Three months follow-up

At the 3-month follow-up, significantly more participants in the intervention arm self-reported that they had increased their activity level during the current sick spell (*p* = 0.033). Still, there was no difference in scheduled activity, symptoms, or coping (data not shown).

### Six months follow-up

There were significantly more participants in the intervention arm self-reporting that they had increased their activity level during the current sick spell (*p* = 0.009). Still, there was no significant change in scheduled activity, symptoms or coping (data not shown).

### Twelve months follow-up

Outcome variables after 12 months are shown in Table 5. After one year, there was no significant difference in net sick leave days between the study arms although significantly more patients in the intervention arm still reported that they had increased their activity level (*p* = 0.018).

**Table 4.** Baseline values for participants in the LEARN-to-COPE cluster randomized controlled study.

Outcome variable	Total *n = 243*	Control *n = 142*	Intervention *n = 101*	*p*-Value
Self-reported sick leave and health-related unemployment				
On sick leave, *n* (%)	187 (85.4)	110 (82.7)	77 (89.5)	0.16
Full-time sick leave, *n (%)*	127 (67.9)	81 (73.6)	46 (59.7)	0.14
Part-time sick leave 75%, *n* (%)	8 (4.3)	5 (4.5)	3 (3.9)	
Part-time sick leave 50%, *n* (%)	43 (23.0)	21 (19.1)	22 (28.6)	
Part-time sick leave 25%, *n* (%)	9 (4.8)	3 (2.7)	6 (7.8)	
Self-assessed increase in activity level during current sick spell, *n* (%)	105 (56.1)	67 (60.9)	38 (49.4)	0.12
Participation in rehabilitation (self-reported)				
Any rehabilitation, *n* (%)	90 (48.1)	54 (49.1)	36 (46.8)	0.75
Medical rehabilitation, *n* (%)	70 (28.8)	41 (28.9)	29 (28.7)	0.98
Weekly hours, mean (SD)	3.0 (4.5)	3.4 (5.7)	2.4 (1.3)	0.27
Work-oriented rehabilitation, *n* (%)	33 (13.6)	22 (15.5)	11 (11.0)	0.32
Weekly hours (independently of sick leave), mean (SD)	5.1 (5.8)	5.7 (5.5)	4.3 (6.5)	0.62
Sense of coherence, mean (SD)	51.4 (14.1)	52.3 (15.1)	50.0 (12.3)	0.25
Comprehensibility, mean (SD)	18.6 (6.2)	19.1 (6.7)	17.9 (5.3)	0.12
Manageability, mean (SD)	15.5 (4.8)	15.7 (5.0)	15.2 (4.5)	0.51
Meaningfulness, mean (SD)	17.3 (5.0)	17.5 (4.9)	17.0 (5.1)	0.47
Health literacy, mean (SD)	12.0 (3.5)	11.9 (3.7)	12.1 (3.2)	0.72
Inadequate, *n* (%)	36 (16.4)	22 (16.5)	14 (16.3)	0.96
Problematic, *n* (%)	74 (33.8)	44 (33.1)	30 (34.9)	
Sufficient, *n* (%)	109 (49.8)	67 (50.4)	42 (48.8)	
Health-related quality of life, mean (SD)	0.43 (0.3)	0.42 (0.33)	0.45 (0.33)	0.48
Symptoms of mental illness				
Depression, mean (SD)	21.0 (10.0)	20.9 (10.4)	21.2 (9.3)	0.84
Exhaustion, mean (SD)	38.2 (9.6)	37.9 (9.7)	38.6 (9.4)	0.62
Anxiety, mean (SD)	16.0 (5.6)	15.7 (5.8)	16.4 (5.1)	0.35
Pain				
Risk of long-term sick leave due to pain, *n* (%)	113 (59.5)	73 (63.5)	40 (53.3)	0.16
Widespread pain, *n* (%)	89 (40.6)	54 (40.6)	35 (40.7)	0.99
Pain catastrophizing >30, *n* (%)	51 (21.3)	30 (21.1)	21 (21.6)	0.92
Lifestyle habits				
Sedentary lifestyle, *n* (%)	44 (20.1)	20 (15.0)	24 (27.9)	0.051
Alcohol high, *n* (%)	4 (1.8)	3 (2.3)	1 (1.2)	0.56
Smoking (yes + sometimes), *n* (%)	44 (20.1)	26 (19.5)	18 (20.9)	0.80
Obesity, BMI ≥ 30, *n* (%)	74 (34.9)	48 (37.8)	26 (30.6)	0.28
Perceived work ability, mean (SD)	2.9 (2.7)	2.8 (2.7)	2.9 (2.6)	0.76
Self-efficacy / confidence in own abilities, mean (SD)	46.0 (13.1)	46.4 (13.5)	45.4 (12.5)	0.58

Missing values not included. *p*-Values indicate tests of differences between intervention and control groups.

Self-assessed increase in activity level during current sick spell: Self-assessed change in activity level was assessed with the question ‘Have you increased your activity level during current sick spell? Yes/No’; Sense of coherence: Sense of Coherence Scale-13 (SOC-13); Health Literacy: European Health Literacy Survey Questionnaire, 16-item version, Swedish edition (HLS-EU-Q16-SE); Health-related quality of life: EuroQol 5-Dimension (EQ-5D); symptoms of depression: Montgomery-Åsberg Depression Rating Scale - Self-rated version (MADRS-S); symptoms of exhaustion: Karolinska Exhaustion Disorder Scale (KEDS); symptoms of anxiety: Generalized Anxiety Disorder 7-item Scale (GAD-7); The risk of future disability and long-term sick leave because of pain: Örebro Musculoskeletal Pain Screening Questionnaire (ÖMPSQ); Widespread pain was defined as pain registered in both sides of the body, above and below the waist, and in the axial skeleton; Pain catastrophizing: Pain Catastrophizing Scale (PCS); A sedentary lifestyle included extended sitting and minimal exercise (not significantly elevating the breathing rate) less than 4 h per week; Hazardous alcohol consumption was defined as 3–4 standard drinks (12 grams of pure alcohol) 2–3 times per week or more; Smoking included both yes and sometimes; BMI: Body Mass Index; Perceived workability: Work Ability Score (WAS) VAS scale; Self-efficacy/confidence in own abilities: study specific questionnaire range 17–85 points, a higher value indicates better self-efficacy.

Juxtaposing registry data and self-reported data for each participant to analyze the change in scheduled activities after 12 months showed that the most common individual outcomes included RTW, waiting for planned rehabilitation to start, became unemployed or lost their sickness benefit but not working, and no change or less scheduled activity. Less common outcomes were participation in rehabilitation, became a student, or early retirement. There was no statistically significant difference in the change of scheduled weekly hours for work or work-related activities.

Notably, a non-negligible proportion of participants was uninsured (at least 26% plus suspected in 9%), and half of the unemployed participants were on registered sick leave (54%).

Linear mixed model analyses revealed statistically significant improvements after 12 months in health literacy and self-efficacy in favor of the intervention group. An improvement in BMI favoring the intervention group (*p* = 0.083) was observed, although it was not statistically significant. Participants in the intervention arm showed greater improvements in sense of coherence, symptoms of mental illness, self-assessed workability, and other lifestyle habits compared to those in the control arm, although these changes were not statistically significant.

Patient-reported quality of care differed significantly between the study arms, with notably fewer participants in the intervention arm reporting good continuity with their GP and rehabilitation coordinator. In all other aspects, there were no significant differences between the study arms. Notably, less than half of the participants overall felt well-informed about available treatment options (36%), the role of the rehabilitation coordinator (40%), and how other healthcare professionals (41%) or authorities (25%) could contribute to their rehabilitation.

## Discussion

The LEARN-to-COPE intervention focused on strengthening participant health literacy and sense of coherence in a social insurance context to improve participants’ ability to collaborate with healthcare and the community to optimize their health and workability. Results showed statistically significant changes in health literacy and self-efficacy favoring the intervention group after 12 months and a general tendency towards more positive outcomes in the intervention arm (sense of coherence, symptoms of mental illness, lifestyle habits, and self-assessed workability).

However, neither the statistically significant improvements in health literacy and self-efficacy nor the overall trend of more positive secondary outcomes in the intervention group were reflected in the primary outcome, i.e. scheduled (work or work-oriented) rehabilitation activities or registry sick leave data.

### Interpretation of results and comparison with other studies

The risk of type I errors increases when examining multiple variables. Consequently, the significant changes in health literacy and self-efficacy may be false positives. Still, because we observed a consistent trend favoring the intervention across most outcomes – and none favoring the control – the overall likelihood of a coincidental positive trend appears low.

Participant work ability at baseline was very poor, with most participants having a history of very long-term sick leave. There was also a substantial proportion of unemployed patients. As the same enrollment process was applied to both the intervention and control groups, a similar inclusion pattern emerged in both arms. We suggest that impaired quality of care, as shown in the study’s contextual data, may hinder healthcare employees’ ability to promptly identify patients requiring more extensive support, consistent with previous findings [41].

Participant’s sense of coherence at baseline was low, as expected, but it did not improve significantly during follow-up, contrary to our hypothesis. However, consistent with previous research [41], few participants experienced relationship continuity with a GP or a rehabilitation coordinator. Moreover, fewer than half felt well-informed about their symptoms or aware of how different healthcare professionals and case workers could assist them.

These findings suggest that addressing a low sense of coherence is important to counteract hopelessness and prevent patients from taking a victim role. However, in line with our previous findings [23,42], we argue that enhancing patients’ perception of life as comprehensible, manageable, and meaningful would require an SRP approach that prioritizes continuity in patient-provider relationships and teamwork, thereby enabling collaborative and creative solutions to overcome individual RTW barriers.

Half of the participants had limited health literacy at baseline, potentially leading to suboptimal health-related decisions. Moreover, a minority of participants felt well-informed about their health conditions and the available SRP options. These findings suggest a pressing need to enhance both patient health literacy and the comprehensibility of the SRP system. These conclusions align with the emerging concept of social insurance literacy [43], which, unlike traditional health literacy, emphasizes the role of system comprehensibility in influencing patient understanding.

Few participants in this study reported participating in work-oriented rehabilitation or receiving extensive medical rehabilitation interventions. Still, despite sparse interventions, very few participants received a disability pension during study follow-up. Unfortunately, our finding of long passive sick leave, few interventions to break the pattern, and seemingly no way out, is in line with previous research [41].

By juxtaposing registry and patient-reported data, we found that participants with prevailing impaired work ability could be falsely presumed to have returned to work when they were no longer registered as being on sick leave. End of sick leave was found to mean ‘no longer receiving sickness benefit’ rather than ‘returned to work’ as individual outcomes in our cohort included ‘no longer part-time sick leave, instead part-time employment’, ‘became unemployed’, and ‘still unemployed, no longer insured’.

Thus, optimizing sick leave key figures as a target may, as a side effect, increase long-term unemployment or subsistence allowance. We conclude that it is important to take person-centered outcomes into account when evaluating the SRP, and that registry data combining information about registry sick leave with health-related and work-related variables is necessary to facilitate such evaluation.

Building on the pilot study conducted before this randomized controlled trial [20], we hypothesized that the LEARN-to-COPE intervention would enhance both the primary outcome, scheduled activity (work or work-oriented rehabilitation), and secondary outcomes within this cohort. Nevertheless, the study had no effect on scheduled activity or sick leave.

Lack of study power may have contributed to the lack of intervention effect. However, the participants reported extensive symptomatology and poor quality of life at baseline, and the duration of their impaired work ability was exceptionally long. Therefore, we conclude that single short-term interventions are unlikely to adequately improve health and work ability in this cohort, regardless of study power. Nevertheless, given that the secondary outcomes generally trended in a more positive direction despite the complexity of participants’ healthcare problems, it is possible that intervening earlier in the SRP - before participants experienced feelings of ‘giving up’ [41] – might have yielded a different result.

This conclusion is supported by the failure of previous SRP intervention research to deliver clear SRP recommendations regarding long-term effective interventions for the target group [9] and research suggesting an unclear association between symptom reduction and sick leave [21]. It is further supported by previous research describing how the SRP entails process-related suffering due to conflicting information, loss of control, harmful passivity due to misapplied sick leave, and non-medical factors driving ill health being overlooked [41].

We suggest that empowering patients to take an active part in SRP-related problem-solving, which was the aim of the LEARN-to-COPE intervention, may be one cog in the machinery, but this cog depends on the SRP for successful process outcomes.

The conditions of the RCT were different from the pilot study of the LEARN-to-COPE intervention [20], which was implemented in a local context with well-functioning SRP procedures. In the pilot, patients discussed eligible options with their GP after participating in the intervention, and there were established ways of collaboration with the Social Insurance Agency, the Employment Agency, and the Social Services if needed. This suggests that SRP interventions may be successfully centralized, but SRP outcomes are dependent on local procedures for individual assessments and follow-up recommendations, consistent with implementation research [44].

Based on the compiled findings from this study and previous research [23,41,42], we argue that addressing the complex burden of symptoms in the target group will require a holistic system approach rather than single short-term interventions. Therefore, we recommend that future improvement efforts and research focus on optimizing basic prerequisites for good care: creating consensus around understanding complex health problems [23] and person-centered organizational priorities to enable individualized care [42].

Indeed, a variety of interventions to solve patients’ medical and non-medical problems will be needed in the SRP toolbox. However, we believe that starting with evaluating interventions or collaborations in a flawed context, rather than mending the SRP process as a whole, entails a risk of rejecting tools and collaborations that could be useful. It also directs focus away from addressing process-related issues.

In line with previous research [45], we found that coordination benefits from concentrating the responsibility for providing the intervention to a few persons. However, centralization did not impact the local procedure for selecting participants, attendance, or the quality of follow-up recommendations. Nevertheless, the feasibility of collaborations between primary healthcare and educational associations - leveraging primary healthcare professionals’ knowledge of the target group while avoiding the use of healthcare resources for interventions that do not require medical expertise - warrants further evaluation.

### Strengths and limitations

The cluster randomized controlled trial design enabled participant recruitment and intervention delivery across geographically dispersed PCCs.

The pragmatic inclusion criteria - based on real-life SRP needs regardless of diagnosis, unemployment status, or duration of sick leave - added to the originality of this primary healthcare study. It was also a key strength that we manually analyzed individual cases to understand outcomes beyond registry sick leave data.

The practical procedures, with enrollment of participants being coordinated by the local rehabilitation coordinators in dialogue with each patient’s GP, were co-created together with on-site healthcare employees to increase study feasibility through streamlined processes. Allowing on-site healthcare employees to select participants provided insights into the barriers to large-scale implementation in real-life settings, which was a strength of our design.

However, while the pragmatic enrollment process allowed for professional judgement as to whether patients were likely adhering to an existing RTW plan and thus aimed to reflect real-world decision-making complexity and broaden the range of eligible patients, in practice it resulted in only including patients with extremely prolonged work disabilities. Our results indicated impaired quality of care, thus obstructing evaluation of the LEARN-to-COPE intervention in adequate settings.

Moreover, our pragmatic inclusion criteria, enrollment procedures, and manual analysis of outcomes led to a large variation in how many participants were included from the various participating primary healthcare centers. Moreover, manual analysis involved an element of interpretation although the analysis was done without knowledge of participant allocation, and there were more participants lost to follow-up than was anticipated, which affected the study’s power.

Notably, the intervention was given during working hours, presupposing continued sick leave or unemployment to participate (8 weeks delay). In the control arm, there was no process-related waiting. The authors found no practical solution to compensate for this potential source of bias. Swedish regulations regarding preventive sick pay apply only to medical treatments and rehabilitation [46]. Unfortunately, there was no exception for research subjects.

The Covid pandemic compromised the quality of this study, which was conducted from 2019 to 2021. Fear of spreading the infection affected the attrition rate in the intervention arm and reduced attendance at group meetings. Additionally, access to work-oriented rehabilitation efforts declined during the pandemic.

The LEARN-to-COPE intervention did not require healthcare expertise, which provided the opportunity for implementation in close collaboration with region-wide educational associations. Evaluating the implementation of the intervention across geographically dispersed PCCs in collaboration with educational associations, alongside assessing the intervention itself, was a notable strength.

## Conclusion

This cluster randomized controlled study showed that the LEARN-to-COPE intervention could significantly improve participants’ health literacy and self-efficacy but not their sense of coherence or scheduled (work or work related) activity. Considering the complex burden of symptoms in the target group, the focus of future research and improvement efforts should be on improving the SRP as a whole rather than on looking for quick solutions to the patients’ complex health problems. Centralized implementation of interventions in close collaboration between primary healthcare and community actors was a promising concept that deserves further evaluation.

## Supplementary Material

SI 1 Self_efficacy_confidence in own abilities 240629.pdf

SI 2 Quality of care 240629.pdf
