# Supplementary material for: Effect on work ability and health-related quality of life following an interactive patient education aiming to increase sense of coherence and health literacy – the LEARN-to-COPE cluster randomized trial
Source: Scand J Prim Health Care. 2025 May 22;43(4):805–20. doi: 10.1080/02813432.2025.2507859 (PMC12632209; doi:10.1080/02813432.2025.2507859)
Supplement: SI 1 Self_efficacy_confidence in own abilities 240629.pdf [file IPRI_A_2507859_SM0961.pdf]

S 1: Patient-reported self-efficacy (confidence in own abilities) after 12 months

(Participants provided answers for each statement on a 5-level Likert scale that ranged from not at all true to completely true.)

How confident are you that you know…

- What you could do to feel better?
- What you can do if you have trouble sleeping (e.g., sleep too much or too little)?
- When you should contact or visit a healthcare provider regarding your illness/symptoms?
- How to get staff at the health center to understand your problems?

How confident are you that you can…

- Maintain your usual social activities?
- Maintain your usual activities at home?
- Maintain your usual activities outside the home?
- Overcome obstacles and focus on opportunities?
- Handle difficult or new tasks?
- Cope with setbacks in specifically demanding situations?
- Get professional emotional support if you need it?
- Manage feelings of loneliness?
- Get information about your illness/symptoms from the health center?

How confident are you that you can do things that alleviate symptoms of ill health?

- If you were to feel depressed - can you then influence your feeling by becoming active?
- If you were to feel anxiety - can you then influence your feeling by becoming active?
- If you were to feel stressed - can you then influence your feeling by becoming active?
- If you think of yourself as inferior or not good enough - can you then influence the thought by becoming active?
