# Supplementary material for: Effect on work ability and health-related quality of life following an interactive patient education aiming to increase sense of coherence and health literacy – the LEARN-to-COPE cluster randomized trial
Source: Scand J Prim Health Care. 2025 May 22;43(4):805–20. doi: 10.1080/02813432.2025.2507859 (PMC12632209; doi:10.1080/02813432.2025.2507859)
Supplement: SI 2 Quality of care 240629.pdf [file IPRI_A_2507859_SM0960.pdf]

S 2: Patient-reported quality of care after 12 months

(Participants provided answers for each statement on a 5-level Likert scale that ranged from not at all true to completely true.)

How well do the statements below correspond to your situation?

[Person-centered information]

- I have received good information about which doctor is responsible for my treatment.
- I have received good information about my illness/my symptoms.
- I have received good information about the available treatment options.
- I have received good information about the medications I have been prescribed, so that I understand their effects and how they should be taken.
- I have received good information about the role of the rehabilitation coordinator.
- I have received good information about what other healthcare professionals can contribute to my rehabilitation.
- I have received good information about what other authorities can contribute to my rehabilitation.

[Relationship continuity]

- I have seen the same doctor throughout the entire study period.
- I have seen the same rehabilitation coordinator throughout the entire study period.

[Quality of relationships]

- The doctor has been respectful towards me.
- The doctor has shown commitment and cared about me.
- The rehabilitation coordinator has been respectful towards me.
- The rehabilitation coordinator has shown commitment and cared about me.
- Other healthcare professionals have been respectful towards me.
- Other healthcare professionals have shown commitment and cared about me.
- Officials from other authorities have been respectful towards me.
- Officials from other authorities have shown commitment and cared about me.

[Involvement in health-care decisions]

- I have (possibly together with healthcare and other authorities) been involved in developing an action plan for my care and rehabilitation.
- I feel involved in decisions regarding my treatment.
- I believe that I increased my opportunities to achieve individual goals through my action plan/goal work
